# Manual therapy versus advice to stay active for nonspecific back and/or neck pain: a cost-effectiveness analysis

**DOI:** 10.1186/s12998-022-00431-7

**Published:** 2022-05-16

**Authors:** Emmanuel Aboagye, Stina Lilje, Camilla Bengtsson, Anna Peterson, Ulf Persson, Eva Skillgate

**Affiliations:** 1 Unit of Intervention and Implementation Research for Worker Health, Institute of Environmental Medicine, Karolinska Institutet, Stockholm, Sweden; 2 Department of Occupational Health Sciences and Psychology, Centre for Musculoskeletal Research, University of Gävle, Gävle, Sweden; 3 Department of Health Promoting Science, Musculoskeletal and Sports Injury Epidemiology Center, Sophiahemmet University, Stockholm, Sweden; 4 The Swedish Institute for Health Economics, Lund, Sweden

**Keywords:** Back and neck pain, Cost-effectiveness analysis, QoL, Pain intensity, Manual therapy

## Abstract

**Background:**

Low back and neck pain are the most common musculoskeletal disorders worldwide, and imply suffering and substantial societal costs, hence effective interventions are crucial. The aim of this study was to evaluate the cost-effectiveness of manual therapy compared with advice to stay active for working age persons with nonspecific back and/or neck pain.

**Methods:**

The two interventions were: a maximum of 6 manual therapy sessions within 6 weeks, including spinal manipulation/mobilization, massage and stretching, performed by a naprapath (index group), respectively information from a physician on the importance to stay active and on how to cope with pain, according to evidence-based advice, at 2 occasions within 3 weeks (control group). A cost-effectiveness analysis with a societal perspective was performed alongside a randomized controlled trial including 409 persons followed for one year, in 2005. The outcomes were health-related Quality of Life (QoL) encoded from the SF-36 and pain intensity. Direct and indirect costs were calculated based on intervention and medication costs and sickness absence data. An incremental cost per health related QoL was calculated, and sensitivity analyses were performed.

**Results:**

The difference in QoL gains was 0.007 (95% CI − 0.010 to 0.023) and the mean improvement in pain intensity was 0.6 (95% CI 0.068–1.065) in favor of manual therapy after one year. Concerning the QoL outcome, the differences in mean cost per person was estimated at − 437 EUR (95% CI − 1302 to 371) and for the pain outcome the difference was − 635 EUR (95% CI − 1587 to 246) in favor of manual therapy. The results indicate that manual therapy achieves better outcomes at lower costs compared with advice to stay active. The sensitivity analyses were consistent with the main results.

**Conclusions:**

Our results indicate that manual therapy for nonspecific back and/or neck pain is slightly less costly and more beneficial than advice to stay active for this sample of working age persons. Since manual therapy treatment is at least as cost-effective as evidence-based advice from a physician, it may be recommended for neck and low back pain. Further health economic studies that may confirm those findings are warranted.

*Trial registration* Current Controlled Trials ISRCTN56954776. Retrospectively registered 12 September 2006, http://www.isrctn.com/ISRCTN56954776.

**Supplementary Information:**

The online version contains supplementary material available at 10.1186/s12998-022-00431-7.

## Background

Low back pain is one of the most common health problems, with a lifetime prevalence of 84%, mainly striking women and those aged 40–80 years [1, 2]. Low back pain has rapidly, in just a decade, been ranked as one of the leading causes of disability and is currently affecting around 83 million people worldwide [3, 4]. Neck pain is also a very common health disorder, especially in women. The one-year prevalence was 25% among women and 16% among men peaking in individuals aged 30–59 years in a study of the general population of Stockholm, Sweden [5]. Neck pain is one of the main causes of disability throughout the world and requires greater attention from governments, health care providers and researchers [6, 7].

Beside physical and social suffering for the individual, back and neck pain also lead to substantial societal costs, but despite increased health expenditures, no corresponding improvement in self-assessed health are observed [8]. In Sweden, musculoskeletal disorders have been estimated to comprise 33% of the total health insurance costs [9].

Back and neck pain are also among the most common reasons for seeking primary health care [7, 8, 10], and guidelines for these problems include strategies such as advice to promote self-management and physical activity [11]. A systematic review concluded that therapies that involve manual therapy (MT) and exercises for neck pain were the most effective [12], and previously reported results from the trial of naprapathic manual therapy on back and neck pain that the present study is based on, support the effectiveness of MT both in the short [13] and in the long run [14]. Recent published research about interventions for back and neck pain is heterogeneous in terms of for example interventions, control condition and setting [15–18], which makes comparisons between studies difficult [19], and researchers have concluded that future studies should include economic evaluations [20, 21].

A Swedish study found that the indirect costs for low back pain were substantially higher than the direct costs, indicating that more effective prevention of chronic low back pain could lead to cost savings even if the treatment costs are higher [22]. Another Swedish health economic study based on a trial on common musculoskeletal disorders other than back and neck pain performed in specialized care (on orthopedic outpatients) showed lower mean cost per patient and larger improvement from MT than from orthopedic standard care [23]. A Dutch study found that MT for neck pain was more effective and less costly when compared with physiotherapy or care by a general practitioner, in primary care [24]. The findings were supported by a systematic literature review, but the authors stated that more high-quality research is needed to make firm conclusions about the use of MT as a cost-effective treatment in clinical practice [25]. To the authors’ knowledge only one published health economic study found no advantages in health improvement, costs, or recurrence rate for MT [26].

Evaluations of therapeutic interventions for back and neck pain should provide information not only of the effectiveness, but also the cost-effectiveness (i.e., costs and quality of life (QoL) and pain intensity measures) to inform not only patients, but also health care providers and policy makers [27, 28]. There are some published health economic studies on MT for musculoskeletal pain, but more studies are warranted to increase the evidence base. The aim of this study was to evaluate the cost-effectiveness of MT compared with evidence-based care from a general practitioner; advice to stay active (ASA), for persons of working age with nonspecific back and/or neck pain. The study was performed from a societal perspective.

## Methods

Data from a randomized controlled trial (RCT) with12 months follow-up of the BJORN trial were used. The trial was approved by the Ethics Committee of the Karolinska Institutet (Diary number 03-657) and registered in a public registry (Current Controlled Trials ISRCTN 569 54776).

### Setting and participants

The BJORN-trial is extensively described previously [13, 14]. Briefly, it is an RCT comprising 409 persons with non-specific pain and disability in the back and/or neck, recruited at two large public companies (n = 40,000) in Stockholm, Sweden. Inclusion criteria were pain and disability in the back and/or neck that brought about marked dysfunction at work or at leisure and had lasted for at least 2 weeks. The two interventions were MT, including spinal manipulation/mobilization, massage and stretching (index group), and ASA; information on the importance to stay active and on how to cope with pain, according to the best scientific evidence available (control group). The advice on staying active was general. The MT group received a maximum of six treatments (within six weeks), by one of eight manual therapists (naprapaths). Naprapaths are a licensed group of manual therapists common in the Nordic countries, and the treatment was a combination of manual techniques such as spinal manipulation and mobilization, and soft tissue techniques such as massage and stretching, in combination with home exercises. The treatments and the advice on home exercise and ergonomics were adapted to each person’s condition. The control group received ASA provided by physicians in direct conjunction with the medical examination that all study participants had at baseline, before inclusion in the trial. A second consultation with a physician was scheduled after 3 weeks, and additional consultations were offered if necessary.

### Measurements

All outcomes were self-rated by repeated postal or web-based measurements questionnaires up to one year after inclusion. The primary outcomes were a clinically meaningful improvement in pain intensity and pain related disability [29], calculated by comparing the results of measurements with the Chronic Pain Questionnaire (CPQ) [30], with three items on pain and three on disability [31, 32], with numerical 11-point rating scales [33, 34] for each participant. A pain score was constructed from the mean of the three pain items and a disability score from the mean of the three disability items. For the analysis in this study, effectiveness measures from the one-year follow-up were used. The Short Form-36 Health Survey SF-36 [35] was used to calculate Quality of Life (QoL). A condensed version with 6 out of the 8 dimensions from the SF-36 (the SF-6D) contains an algorithm which makes it possible to derive QoL [36]. Further, the change in pain intensity from baseline to one-year follow-up was used.

### Cost calculation

Direct costs included costs for interventions and drugs from “Prices and compensations for the health care region of Stockholm/Gotland”, from Karolinska Hospital. The question about health care consumption and use of medication were included in the questionnaires and read: “Please report which of the health care/investigation options you sought for the disorders of the neck/back in the last 6 months, in addition to what was included in this trial”. Answer alternatives: (1) I have not sought any health care/investigation, (2) Physiotherapist, (3) Naprapath, (4) Chiropractor, (5) Massage therapist, (6) Medical doctor, (7) Investigations such as radiography or similar, (8) Other. The participants were also asked to specify the number of visits for each alternative. As regards the use of medication, the participants were asked whether they had taken medications/natural supplements because of back/neck pain, and if so, how often. The costs were valued using prices provided by the pharmacy. Indirect costs included costs of production loss due to sick leave. Information on salaries for different occupations were calculated according to Statistics Sweden (SCB). The production loss was calculated as number of sick leave days/365 * yearly salary * 1.41 which is the average social fee for workers in Sweden, using the human capital approach [37].

All costs were collected in Swedish crowns (SEK) for the year 2005 (which corresponds to the period in which the RCT was conducted) and adjusted for inflation using the consumer price index, to reflect prices for the year 2019/20. The exchange rate used to convert costs into EUR was 1 SEK = 9.29 EUR. No discounting was applied since the interventions and the follow-ups occurred within one year.

### Statistics

The societal perspective on the impact of resource use was followed when conducting the economic evaluation. This implies that all relevant costs and effects of the interventions related to non-specific back and neck pain in participants are considered, regardless of who pays or benefits. Data from the participants who withdrew from the trial were used until the time of withdrawal. Costs and utilities were analyzed within a one-year horizon, since the RCT followed participants for one year.

In the main analysis, a cost-effectiveness analysis was performed only on participants with complete data on costs and QoL to determine if the intervention resulted in larger effects than the alternative, and at what additional cost, in terms of incremental cost-effectiveness ratio (ICER) (i.e., dividing the incremental costs by the incremental Quality of Life (QoL)). The economic value of providing an intervention was represented by the ICER. The differences in costs and QoL between interventions are visually represented in a cost-effectiveness plane by using the percentile bootstrapping method with 5000 replications of total costs and QoL.

For decision-making purposes, the ICER is useful when the intervention is more costly but generates improved health effect. In such cases there is a need to compare the ICER with a pre-determined threshold to decide whether choosing the intervention is an efficient use of resources. Another method is to use the net benefit approach and generate acceptability curves. A cost-effectiveness acceptability curve (CEAC) was used in the present study, to depict the probability that the intervention will be cost-effective at different willingness to pay (WTP) thresholds. A net monetary benefit (NMB) represents the value of an intervention in monetary terms, and a WTP threshold for a unit of benefit (the incremental benefit multiplied by the WTP threshold less the incremental cost) was also calculated.

The statistical analysis described above was repeated for the outcome “change in pain intensity” derived from the Chronic Pain Questionnaire (CPQ) (30). All analyses were performed using Microsoft Excel and SPSS.

### Sensitivity analysis

The robustness of the findings was assessed by performing sensitivity analyses. One sensitivity analysis was performed by using multiple imputations to impute missing data on costs and QoL in both groups, according to the Fully Conditional Specification by a Linear regression method. In the multiple imputation procedure, five imputed data sets were created. The Output Management System (OMS) utility in SPSS was applied to produce pooled estimates of means, standard errors and 95% confidence intervals (CI) of variables of interest in the two groups. In this study, we were interested in the effect of QoL from being allocated to the index group considering the cost or resources required to yield a change in QoL. Thus, we explored the impact of the missing values of study participants behaving like those in the index group in the two arms of the trial on the results in the sensitivity analysis. We performed a reference-based imputation which draws imputed values with some reference to observed data in the other group of the trial.

More generally, our statement about unobserved patient data is that they were missing at random. Therefore, the marginal distribution of the unobserved data will be the same as the conditional distribution of the unobserved data given the observed data will be the same, regardless of whether the data was observed or not. Based on the difference in mean cost and QoL per person between the index group and the control group, we could say that the claim was respected since the results from the sensitivity analysis show no appreciable differences from the main analysis.

Another sensitivity analysis was performed by excluding the costs for participants whose total costs were exceeding three standard deviations of the average costs for the treatments in both arms. Some participants had to undergo surgery and other expensive treatments during the trial period, which supported the decision for conducting a sensitivity analysis based on exclusion of these participants. An ICER and a CEAC were also obtained using the same procedures as in the main analysis.

## Results

**Fig. 1:**
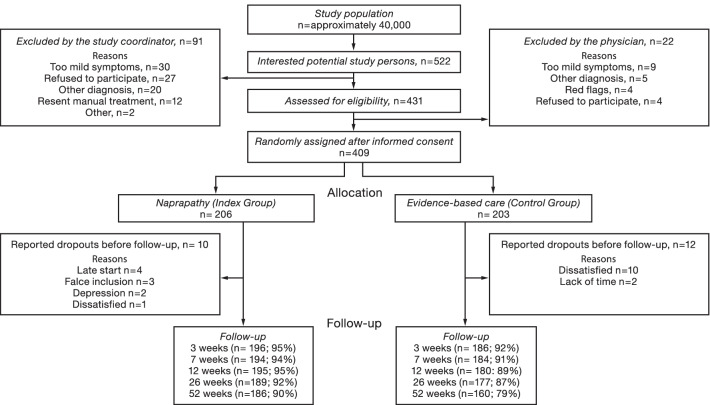
The flow of participants through each stage of the trial and details about dropouts

A total of 409 study participants were randomized to “advice to stay active” group (n = 203) or manual therapy group (n = 206) and followed for 12 months. The participants had a mean age of 47 years, were mainly women (71%), and were mainly suffering from neck pain (58%). Eight percent in the MT group and 6% in the control group suffered from both back and neck pain (13), and for 56% the duration of pain was more than a year [13]. The flow of participants through each stage of the trial is shown in Fig. 1.

However, not all the randomized patients completed the 12-month follow-up. This implies for our complete data analysis a total of n = 40 index group participants and n = 31 control group participants were missing data due to not completing the questions related to the variables used here or withdrawal from the trial. The number of participants at follow-up with any data on costs and QoL used in the complete data analyses used n = 160 for the index group and n = 172 for the control group, even though fewer in the control group were followed for one year. For the pain intensity, the complete data analyses used n = 182 for the index group and n = 158 for the control group. More details about dropouts are reported in a previous publication [14]. There were improvements in pain intensity and pain related disability within both groups compared with baseline, and there were significant differences in clinically meaningful improvements between the groups favoring the index group at 12 months follow-up [13, 14].

### Costs

Prices for each health care intervention are provided in Table 1.

**Table 1:** Prices for each health care intervention in the one-year follow-up of an RCT on MT versus ASA for working age persons with neck and/or low back pain. Price level 2005

Interventions	Price EUR	Price SEK	Interventions	Price EUR	Price SEK
MT	57	450	Bone density	127	990
Physician	179	1400	Gastroscopy	258	2010
Physiotherapist	64	500	Nerve blockade	285	2227
Chiropractor	48	375	Psychologist	139	1083
Massage therapy	51	400	Drugs, prescription		
Medical investigation	75	591	Daily	3.28	25.6
Acupuncture	54	425	Sometimes	0.65	5.12
Oral physiology	256	2000	Drugs, no prescription		
Herniated disc surgery	7965	62,040	Daily	3.63	28.3
Cortisone injection	179	1400	Sometimes	0.73	5.7
NMT* at student clinic	23	180	Herbal remedies		
Neurological examination	128	1000	Daily	8.72	67.9
Pain clinic	159	1245	Sometimes	1.75	13.6

Costs were calculated from baseline until the one-year follow-up. The mean individual cost of care included in the RCT, and self-elected “other treatment” was lower for the index group (600 EUR) compared with the control group (862 EUR) (mean difference = − 262 EUR, 95% CI − 491 to − 33; *p* = 0.02). The costs for prescribed medication were also lower in the index group compared with the control group; 3 EUR in the index group and 6 EUR in the control group (mean difference = − 3 EUR, 95% CI − 4 to − 1; *p* = 0.001). One of the largest contributors to the costs of the groups was production loss due to sickness absence. In total, the index group had 847 sick leave days, and the control group 1395 sick leave days. The mean cost for sick leave days was also lower in the index group (533 EUR), compared with the control group (1037 EUR), mean difference = − 504 EUR, 95% CI − 1285 to 278; *p* = 0.21

Concerning the QoL outcome, the mean total cost (i.e., health care, “other treatments”, medication, and sick-leave) for the groups were 1137 EUR (95% CI 751–1656) for the index group and 1574 EUR for the control group (95% CI 1270–2610), and the difference in mean cost per person between the index group and the control group was  − 437 EUR (bootstrap 95% CI − 1302 to 371), *p* = 0.32. For the pain outcome, the results show that the mean costs for the index group was 1227 EUR (95% CI 752–1702) and 1863 EUR for the control group (95% CI 1078–2648), and the difference in mean cost per person between the index group and the control group was − 635 EUR (bootstrap 95% CI − 1587 to 246), *p* = 0.163.

### Quality of life and pain intensity

The utility gains per person over one year were measured as the changes of QoL from baseline to the one-year follow-up. The mean difference was 0.007 (bootstrap 95% CI − 0.010 to 0.023). The difference between the groups observed for the change in pain intensity between baseline and the one-year follow-up was 0.6 (bootstrap 95% CI 0.068–1.065).

### Incremental cost-effectiveness ratio (ICER)

**Fig. 2:**
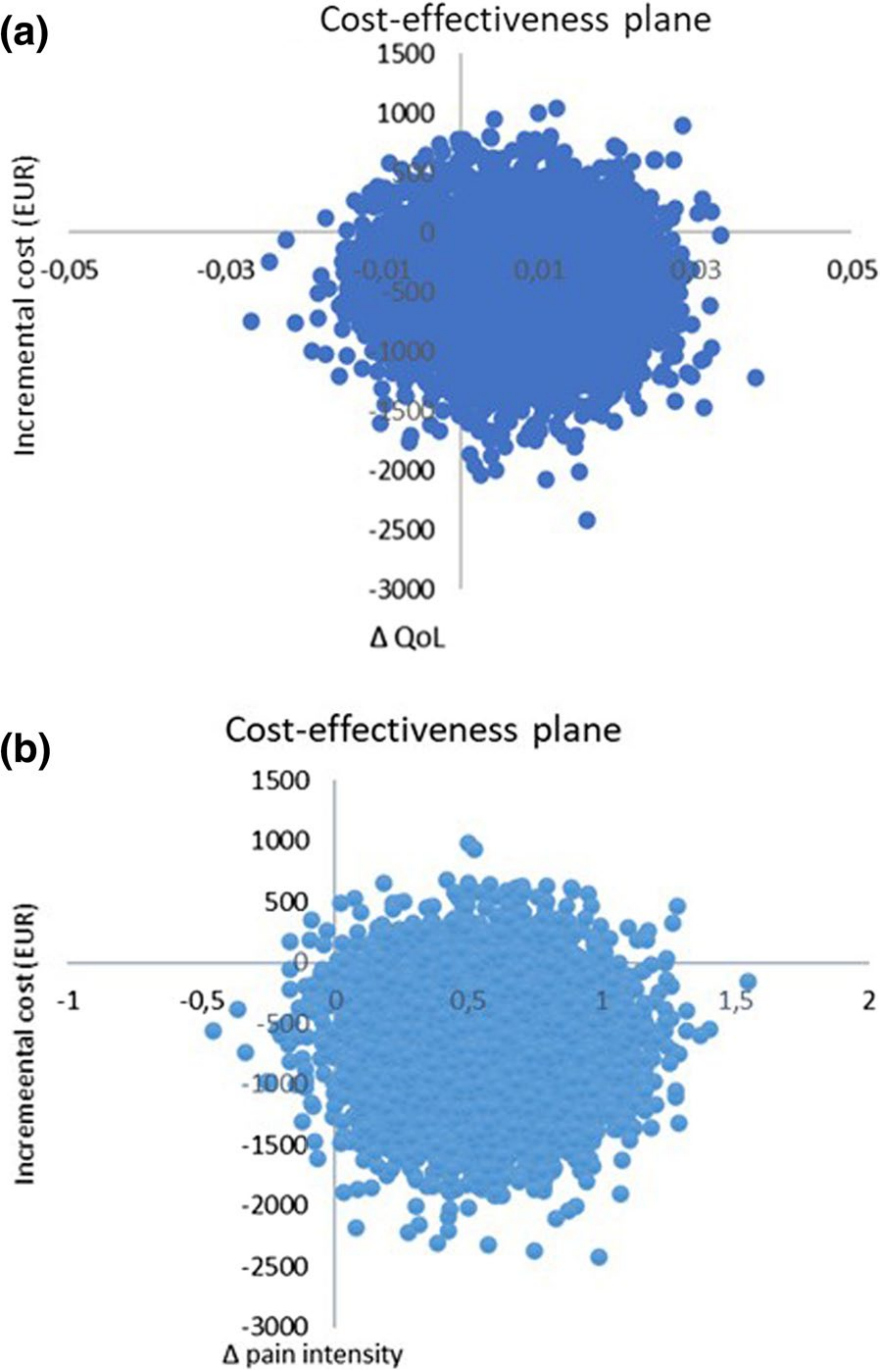
**a**, **b** Cost-effectiveness plane using bootstrapped incremental cost-effectiveness ratios for QoL and pain intensity outcomes

The ICER for the QoL as well as for the pain intensity between the MT and ASA was negative (Fig. 2a). This implies that MT is a dominant treatment option, i.e., it is both less costly and results in better health related QoL and improves pain intensity than ASA. The cost-effectiveness plane for the QoL outcome showed that 12% of the bootstrapped difference between cost and effect units lay in the upper right quadrant (more costly and more effective), 68% indicated dominance (less costly and more effective) in favor of MT, 3% indicated that MT was dominated by ASA and 17% indicated lower costs and less effectiveness for ASA.

The ICER for the pain intensity outcome shows that MT is a cost-effective and dominant treatment option, i.e., it is both less costly and improves pain intensity more than ASA (Fig. 2b). The cost-effectiveness plane showed that 8% of the bootstrapped difference between cost and effect units lay in the upper right quadrant (more costly and more effective), 91% indicated dominance (less costly and more effective) in favor of MT, 0.08% indicated that MT was dominated by ASA, and 0.98% indicated lower costs and less effectiveness for ASA.

A cost-effectiveness plane showing the differences between costs and effect units of MT compared with ASA is shown in Fig. 2a, b.

**Fig. 3:**
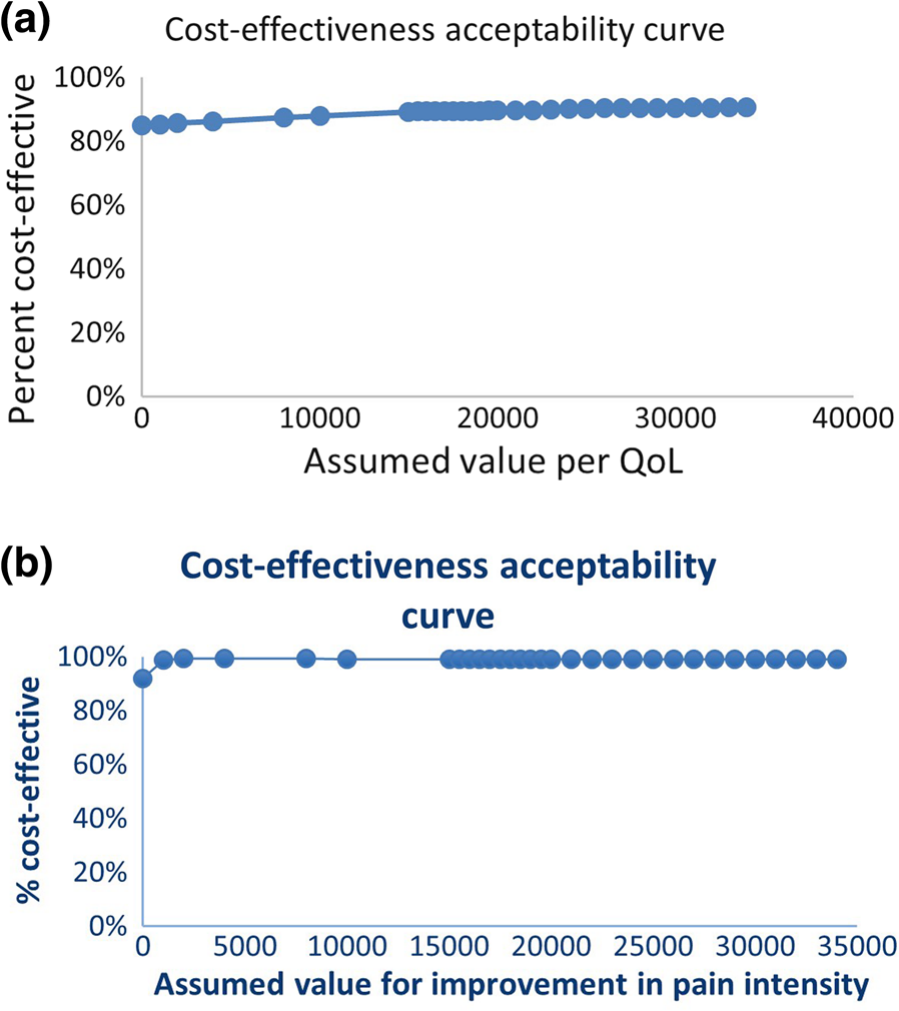
**a**, **b** Cost-effectiveness acceptability curve (CEAC) showing the probability that MT is cost-effective compared with ASA at different WTP thresholds for the QoL and pain intensity outcomes

The cost-effectiveness acceptability curve (CEAC) showed that the probability that MT can be considered cost-effective is about 85% at a zero threshold of willingness to pay per QoL or and 92% for meaningful improvement in pain intensity. At higher thresholds noticeable further increases in the probability of cost-effectiveness of MT may be observed. (Fig. 3a, b).

**Fig. 4:**
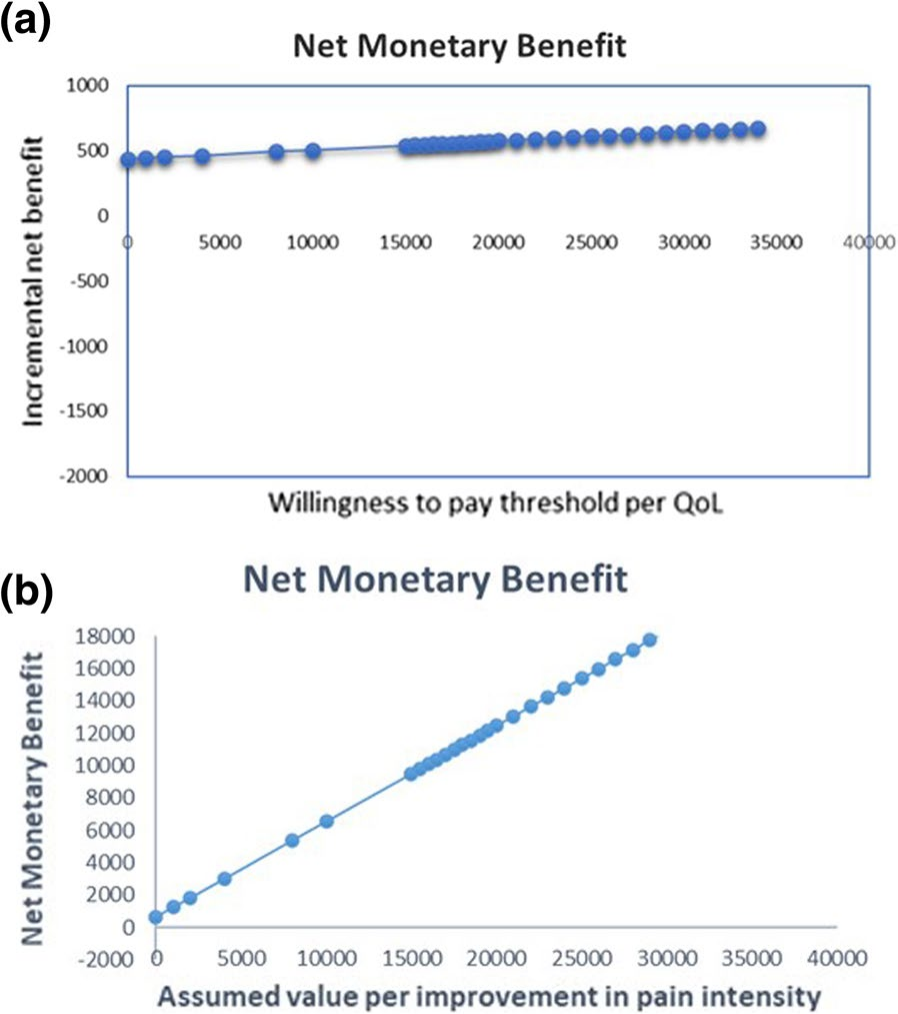
**a**, **b** Net monetary benefit showing the value of MT in monetary terms assuming different willingness to pay thresholds for a unit of benefit, i.e., QoL and improvement in pain intensity. *Note*: WTP thresholds; ΔQ, difference in mean QoL between groups; Δ Pain, improvement in pain intensity; ΔC, difference in mean cost of intervention per group

The Net monetary benefit (NMB) was positive indicating that the MT is cost-effective compared with ASA over a range of the given willingness-to-pay thresholds (Fig. 4a, b). It implies that the cost to derive the benefit is less than the maximum amount that the decision-maker would be willing to pay for this benefit, since the ICERs for QoL and pain intensity was a negative value.

### Sensitivity analysis

When performing multiple imputation (as a sensitivity analysis for the QoL outcome) the mean cost is 1134 EUR in the index group and 1653 EUR in the control group (mean difference -517 EUR (− 1300 to 217), and the mean individual QoL 0.659 in the index group and 0.656 in the control group (mean difference 0.004 for QoL (bootstrap 95% CI − 0.010 to 0.018). When persons with extreme costs of treatment were excluded (12 in total, n = 4 in the index group and n = 8 in the control group) in the sensitivity analysis, the mean cost in the index group decreased from 1235 to 860 EUR, and the mean cost in the control group decreased from 1838 to 1028 EUR. The difference in mean cost was -167 EUR (bootstrap 95% CI − 386 to 53). The difference in QoL was slightly higher in favor of the index group with the difference in QoL between groups being 0.0008 (bootstrap 95% CI − 0.014 to 0.014). Overall, the sensitivity analysis indicates a slightly higher probability for MT being considered cost-effective at lower thresholds of willingness to pay per QoL. Thus, the results show no appreciable differences from the main analysis. Table 2 shows both listwise and imputed cost data used in the sensitivity analysis.

**Table 2:** Listwise and imputed results for the QoL outcome and cost

Variables	Index group (n = 160)		Control group (n = 172)	
	Mean (SD)	95% CI	Mean (SD)	95% CI
*Using only participants with complete data*				
QoL	0.662 (0.079)	0.649–0.675	0.655 (0.074)	0.644–0.667
Sick-leave cost	533 (2908)	191–1010	1037 (4167)	514–1612
Cost of other treatment	600 (662)	517–696	862 (1324)	695–1083
Cost of medication	4 (5)	3–4	6 (8)	5–7

Variables	Index group (n = 206)		Control group (n = 203)	
	Mean (SD)	95% CI	Mean (SD)	95% CI
*Using imputed data for missing values*				
QoL	0.659 (0.073)	0.648–0.669	0.656 (0.073)	0.646–0.667
Sick-leave cost	546 (2807)	236–866	878 (3852)	409–1512
Cost of other treatment	587 (634)	512–674	769 (1239)	625–947
Cost of medication	4 (5)	3–4	5 (8)	4–7

## Discussion

### Summary of findings

In this health economic evaluation of treatments in a working population with nonspecific back and/or neck pain, we found that the gains in QoL and improvement in pain were slightly higher for MT than for ASA, and that both direct and indirect costs were lower for MT. The net monetary benefit was positive indicating less cost to the payer to derive gains from the expenditure relating to the intervention. We have previously, based on the same RCT, shown that MT was clinically and statistically significantly better than ASA as regards pain, physical function, and perceived recovery for back and neck pain both in the short and the long run [13, 14]. The extension of our previous findings of treatment effects with the cost-effectiveness of MT compared with ASA has, to our knowledge, not been investigated in many previous studies on back and neck pain. The strengths and limitations with the analyses of the effects of the interventions are discussed elsewhere [13, 14].

### Strengths and weaknesses

Strengths of the present study include that it is based on a large RCT, which minimizes the risk of confounding from differences in prognostic factors between the groups. The frequency of those (e.g., smoking, education, BMI) was very similar in the groups, meaning a low risk of confounding [13, 14], even though residual and unmeasured confounding cannot be ruled out. The validated SF 36 health survey was used and encoded to SF6D to estimate the QoL, and both direct and indirect costs were measured, which we consider as strengths. Since nonspecific back and neck pain is very common, the results are of importance for a large proportion of the population. To enable evaluation of the two treatments as they are performed in daily practice, the numbers, and lengths of treatments between the groups were allowed to differ, and the content of the interventions was discussed with the health care providers before the start of the trial. This was done to standardize the treatments as far as possible without intruding on the pragmatic design, which we also consider a strength. Further strengths are the intention to treat analyses and relatively few dropouts during the follow-up. However, since the loss-to-follow-up in the index group was larger than in the control group, it may have introduced selection bias. This potential bias would probably not have affected the conclusions of our study though, since the sensitivity analyses performed in the first publication to estimate the impact of missing responses showed no systematic differences in results between analyses with and without imputed primary outcome values [13]. The internal missing of the outcome QoL was larger than the pain intensity outcome resulting in different number of study participants in the analyses. Further limitations should also be addressed. According to the mean cost per person, we divided the total costs in each group by the number of participants at the beginning of the follow-up period. Since the loss-to-follow-up was higher in the control group this might have underestimated the mean cost per person in this group. If, in addition, study participants lost-to-follow-up had higher costs than those who did answer the follow-up questionnaires in the control group, this could potentially also have underestimated the mean cost per person in that group. When multiple imputation was used to perform sensitivity analyses the difference in mean cost per person increased from − 437 to − 603 EUR indicating that this discussion might be right. In summary, the risk would be an underestimation of the differences in costs between the interventions, though, which indicates that the conclusion of our study is valid. A sensitivity analysis was performed regarding participants with extreme costs of treatment, which resulted in cost reductions in both groups, and consistency in the results concerning the costs of the interventions, but not the QoL. This may be considered a weakness, but we think that the main conclusions of our study remain. A final limitation should be addressed. The study was performed in 2005, and therefore we used prices that were valid at the same time. One could argue that prices could have changed, and we also know that sick leave in Sweden has decreased during the last decade. However, this concerns both groups, and since all measures are relative, we believe that our results would apply even if we had performed the study in more recent years.

### Earlier studies

Our results are in accordance with a Swedish study where the indirect costs for low back pain were substantially higher than the direct costs [22], and with a Dutch study that observed that MT was more effective and less costly for treating neck pain than physiotherapy or care by a general practitioner [24]. Further, two systematic literature reviews support our findings [25, 37], although the authors in one of them stated that more high-quality research is needed to make firm conclusions about the use of spinal MT as a cost-effective treatment in clinical practice [25]. In addition, in that review, only one study found no differences in health improvement, costs, or recurrence rate for MT compared with physiotherapy [26]. A previous report from our research group on common musculoskeletal disorders, showed lower mean cost per person and larger improvement of MT (provided by naprapaths) than of orthopedic standard care for common musculoskeletal disorders [23]. However, to our knowledge, our study is the first 12 months follow-up that has investigated the cost-effectiveness of MT compared with ASA for back and/or neck pain. The results in this study are of importance for decision making regarding which care to choose, both from a quality of life and from an economic perspective for a large group of patients.

## Conclusions

Our results indicate that MT is slightly less costly and more beneficial than ASA for working age persons with nonspecific back and/or neck pain. Together with the clinical results from previously published studies on the same population the results suggest that MT may be as cost-effective a treatment as evidence-based advice from a physician, for back and neck pain. Additional health economic studies that may confirm those findings are warranted.

## Supplementary Information

**Additional file 1.** Analysis template: cost-effectiveness analysis of pain intensity.

**Additional file 2.** Analysis template: cost-effectiveness analysis of health-related quality of life (QoL).

## Data Availability

The datasets generated and/or analyzed during the current study are not publicly available due to *General Data Protection Regulation* but are available from the corresponding author on reasonable request.

## Abbreviations

ASA: Evidence-based advice to stay active
CEAC: Cost-effectiveness acceptability curve
CPQ: Chronic Pain Questionnaire
HRQL: Health related quality of life
MT: Manual therapy
NMB: Net monetary benefit
QoL: Quality of life
RCT: Randomized controlled trial
SCB: Statistics Sweden
SF-36: Short Form-36 Health Survey
SPSS: Statistical Package for the Social Science
WTP: Willingness-to-pay

## References

[1] Hoy D, March L, Brooks P, Blyth F, Woolf A, Bain C (2014). The global burden of low back pain: estimates from the Global Burden of Disease 2010 study. Ann Rheum Disord.

[2] Balagué F, Mannion AF, Pellisé F, Cedraschi C (2012). Non-specific low back pain. Review. Lancet.

[3] Buchbinder R, Blyth FM, March LM, Brooks P, Woolf AD, Hoy DG (2013). Placing the global burden of low back pain in context. Best Pract Res Clin Rheumatol.

[4] Murray CJ, Vos T, Lozano R, Naghavi M, Flaxman AD, Michaud C (2012). Disability-adjusted life years (DALYs) for 291 diseases and injuries in 21 regions, 1990–2010: a systematic analysis for the Global Burden of Disease Study 2010. Lancet.

[5] Skillgate E, Magnusson C, Lundberg M, Hallqvist J (2012). The age- and sex-specific occurrence of bothersome neck pain in the general population—results from the Stockholm public health cohort. BMC Musculoskelet Disord.

[6] Hoy D, March L, Woolf A, Blyth F, Brooks P, Smith E (2014). The global burden of neck pain: estimates from the Global Burden of Disease 2010 study. Ann Rheum Disord.

[7] Hogg-Johnson S, van der Velde G, Carroll LJ, Holm LW, Cassidy JD, Guzman J (2008). The burden and determinants of neck pain in the general population: results of the Bone and Joint Decade 2000–2010 Task Force on Neck Pain and Its Associated Disorders. Spine.

[8] Martin BI, Deyo RA, Mirza SK, Turner JA, Comstock BA, Hollingworth W, et al. Expenditures and health status among adults with back and neck problems. JAMA. 2008;299:656–64. Erratum in: JAMA 2008;299:2630. 10.1001/jama.299.6.656 18270354

[9] Vad kostar olika sjukdomar i sjukförsäkringen? Försäkringskassan, March 2011. ISSN 1654-8574.

[10] Luo X, Pietrobon R, Sun SX, Liu GG, Hey L (2004). Estimates and patterns of direct health care expenditures among individuals with back pain in the United States. Spine.

[11] Koes WB, van Tulder M, Ostelo R, Burton AK, Waddell G (2001). Guidelines for the management of low back pain in primary care; an international comparison. Spine.

[12] Chou R, Deyo R, Friedly J, Shelly A, Hashimoto R, Weiner M (2017). Non pharmacological therapies for low back pain: a systematic review for an American College of Physicians Clinical Practice Guideline. Ann Intern Med.

[13] Skillgate E, Vingård E, Alfredsson L (2007). Naprapathic manual therapy or evidence-based care for back and neck pain: a randomized, controlled trial. Clin J Pain.

[14] Skillgate E, Bohman T, Holm LW, Vingård E, Alfredsson L (2010). The long-term effects of naprapathic manual therapy on back and neck pain - results from a pragmatic randomized controlled trial. BMC Musculoskelet Disord.

[15] Walker MJ, Boyles RE, Young BA, Strunce JB, Garber MB, Whitman JM (2008). The effectiveness of manual physical therapy and exercise for mechanical neck pain: a randomized clinical trial. Spine.

[16] Paatelma M, Kilpikoski S, Simonen R, Heinonen A, Alen M, Videman T (2008). Orthopaedic manual therapy, McKenzie method or advice only for low back pain in working adults: a randomized controlled trial with one-year follow-up. J Rehabil Med.

[17] Hoving JL, de Vet HC, Koes BW, Mameren HV, Devillé WL, van der Windt DA (2006). Manual therapy, physical therapy, or continued care by the general practitioner for persons with neck pain: long-term results from a pragmatic randomized clinical trial. Clin J Pain..

[18] UK Beam Trial Team (2004). United Kingdom back pain exercise and manipulation (UK BEAM) randomised trial: effectiveness of physical treatments for back pain in primary care. BMJ.

[19] Driessen M, Chung-Wei L, van Tulder M (2012). Cost-effectiveness of conservative treatments for neck pain: a systematic review on economic evaluations. Eur Spine J.

[20] Furlan AD, Imamura M, Dryden T, Irvin E (2008). Massage for low-back pain. Cochrane Database Syst Rev..

[21] Gross A, Miller J, D'Sylva J, Burnie SJ, Goldsmith CH, Graham N (2010). Manipulation or mobilisation for neck pain: a cochrane review. Man Ther.

[22] Ekman M, Jönhagen S, Hunsche E, Jönsson L (2005). Burden of illness of chronic low back pain in Sweden: a cross-sectional, retrospective study in primary care setting. Spine (Phila Pa 1976).

[23] Lilje SC, Persson UB, Tangen ST, Kåsamoen S, Skillgate E (2014). Costs and utilities of manual therapy and orthopedic standard care for low prioritized orthopedic outpatients of working age: a cost consequence analysis. Clin J Pain.

[24] Korthals-de Bos IB, Hoving JL, van Tulder MW, Rutten-van Mölken MP, Adèr HJ, de Vet HC (2003). Cost effectiveness of physiotherapy, manual therapy, and general practitioner care for neck pain: economic evaluation alongside a randomised controlled trial. BMJ.

[25] Michaleff ZA, Lin CW, Maher CG, van Tulder MW (2012). Spinal manipulation epidemiology: systematic review of cost effectiveness studies. J Electromyogr Kinesiol.

[26] Skargren EI, Carlsson PG, Oberg BE. One-year follow-up comparison of the cost and effectiveness of chiropractic and physiotherapy as primary management for back pain. Subgroup analysis, recurrence, and additional health care utilization. Spine (Phila Pa 1976). 1998;23:1875–83;discussion 1884. 10.1097/00007632-199809010-00016 9762745

[27] Bravo Vergel Y, Sculpher M (2008). Quality-adjusted life years. Pract Neurol.

[28] Shiell A, Donaldson C, Mitton C, Currie G (2002). Health economic evaluation. J Epidemiol Commun Health.

[29] Elliott AM, Smith BH, Smith WC, Chambers WA (2000). Changes in chronic pain severity over time: the chronic pain grade as a valid measure. Pain.

[30] Smith BH, Penny KI, Purves AM, Munro C, Wilson B, Grimshaw J (1997). The chronic pain grade questionnaire: validation and reliability in postal research. Pain.

[31] Underwood MR, Barnett AG, Vickers MR (1999). Evaluation of two time-specific back pain outcome measures. Spine.

[32] Von Korff M, Ormel J, Keefe FJ, Dworkin SF (1992). Grading the severity of chronic pain. Pain.

[33] Farrar JT, Young JP, LaMoreaux L, Werth JL, Poole RM (2001). Clinical importance of changes in chronic pain intensity measured on an 11-point numerical pain rating scale. Pain.

[34] Fejer R, Jordan A, Hartvigsen J (2005). Categorising the severity of neck pain: establishment of cut-points for use in clinical and epidemiological research. Pain.

[35] Sullivan M, Karlsson J (1998). The Swedish SF-36 Health Survey III—evaluation of criterion-based validity: results from normative population. J Clin Epidemiol..

[36] Brazier J, Roberts J, Deverill M (2002). The estimation of a preference-based measure of health from the SF-36. J Health Econ.

[37] SCB. The Swedish Labour Cost Index (LCI). Statistics Central Bureau, Statistics. Sweden; 2021. https://www.scb.se/en/finding-statistics/statistics-by-subject-area/labour-market/wages-salaries-and-labour-costs/labour-cost-index-lci/.

